# Data On Percentage Coral Reef Cover In Small Islands Bunaken National Park

**DOI:** 10.1016/j.dib.2020.105713

**Published:** 2020-05-15

**Authors:** Joshian Nicolas William Schaduw, Khristin Ivone Fisye Kondoy, Victoria Era Nicoline Manoppo, Alfret Luasunaung, Joppy Mudeng, Wilmy E. Pelle, Edwin L.A. Ngangi, Indri S. Manembu, Adnan Sjaltout Wantasen, Deiske Adeliene Sumilat, Natalie D.C. Rumampuk, Sandra Olivia Tilaar, Hermanto W.A. Manengkey, Rosita Lintang, James Yosep Walalangi, Biondi Tampanguma, Faldy Pungus, Yostan Lahabu, Bonke Sagai, Efra Wantah, Letha Louisiana Wantania, Brama Djabar, Aris Putra Oli, Emma Arny Caroles, Fihri Bachmid, Juwinda Sasauw, Ardy Kase, Annice Anthoni, Andrew David Uada, Ringgo Latjandu, Clive Coloay, Sylvia Kojongian, Nadine Nichlen Timothea Mamangkey

**Affiliations:** a Department of Marine Science, Faculty of Fisheries and Marine Science, Sam Ratulangi University, Manado, Indonesia; b Department of Management Aquatic Resources, Faculty of Fisheries and Marine Science, Sam Ratulangi University, Manado, Indonesia; c Departement of Fishery Product Technology, Faculty of Fisheries and Marine Science, Sam Ratulangi University, Manado, Indonesia; d Departement of Aquaculture, Faculty of Fisheries and Marine Science, Sam Ratulangi University, Manado, Indonesia; e Departement of Agribusiness, Faculty of Fisheries and Marine Science, Sam Ratulangi University, Manado, Indonesia; f Department of Management Aquatic Resources, Faculty of Animal Husbandry and Fisheries, Tadulako University, Palu, Indonesia

**Keywords:** Coral Reef, Percent Cover, Small Islands, Bunaken National Park, Underwater Photo Transect, Coral Point Count with excel

## Abstract

This article describes the data on coral reef percent cover small islands (Bunaken Island; Siladen Island; Manado Tua Island; Mantehage Island; Nain Island) in Bunaken National Park, Noth Sulawesi Province, Indonesia. The data of coral percent cover from 20 stations (1000 quadrans), surveys were done by scuba diving along 50 m belt transects established on upper reef slope, mostly with depths ranging from 5 to 10 m. The method Used Underwater Photo Transect (UPT) and Coral Point Count with excel (CPCe) analysis. Percentage of coral cover between 6,27% and 69,20%. Manado Tua Island has a percentage of coral cover 42,02%, Bunaken Island 39,63%, Mantehage Island 29,10%, Siladen Island 27,87%, and Nain Island 25,97%. The average of live coral cover on the five islands is 32.92% with moderate category [2].

**Table:** 

**Subject**	Biology
**Specific subject area**	Marine Biology; Coastal and marine resources management; Marine Conservation
**Type of data**	Table Image Chart Graph Figure
**How data were acquired**	Field visits (Scuba dive), lab based analysis with Coral Point Count with excel (CPCe) [1,2]
**Data format**	Raw Analyzed
**Parameters for data collection**	Coral reef percentage; Life form Coral [1,2]
**Description of data collection**	Underwater photo transeck (UPT) [1,2] Coral Point Count with excel (CPCe) analysis [1,2]
**Data source location**	Small Islands Bunaken National Park, North Sulawesi Province, Indonesia (Bunaken Island; Siladen Island; ManadoTua Island; Mantehage Island; Nain Island)
**Data accessibility**	Data is embedded in this article

**Value of the Data**

• Bunaken National Park in the Coral Triangle area and has a very high biodiversity in the coral reef ecosystem; Bunaken is a very popular diving tourist destination in North Sulawesi; Degradation of coral reefs due to global warming are also indicated on the Bunaken National Park.
• The data can be used by scientist, Bunaken National Park Office; the Education Fund Managing Board of the Ministry of Financial Affairs of Indonesian Republic; the Directorate General of Research and Development Strengthening, the Ministry of Education and Culture of Indonesian Republic; the Ministry of Living Environment and Forestry; the Indonesia Science Institution; the Faculty of Fisheries and Marine Science, Sam Ratulangi University and the local goverment in North Sulawesi province. This data can be used as a baseline in monitoring the Bunaken National Park coral reef in the future.
• This data can be used to develop a more practical and inexpensive method of assessing coral cover in the future.
• This data is useful for people who live in Bunaken National Park in monitoring coral reef conditions, because 95% of the people who live there have a livelihood as fishermen and tour guides.
• The data can be used to create policy for coral reef conservation and coastal management; The data can be used to monitoring and evaluation coral reef condition in Bunaken National Park because observation stations in this study is permanent.

## 1 Data Description

**Fig. 1:**
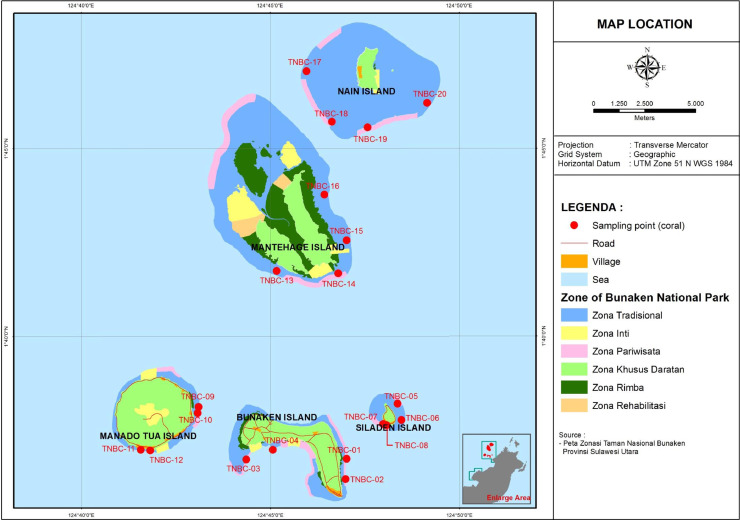
Research Location and sampling point.

**Fig. 2:**
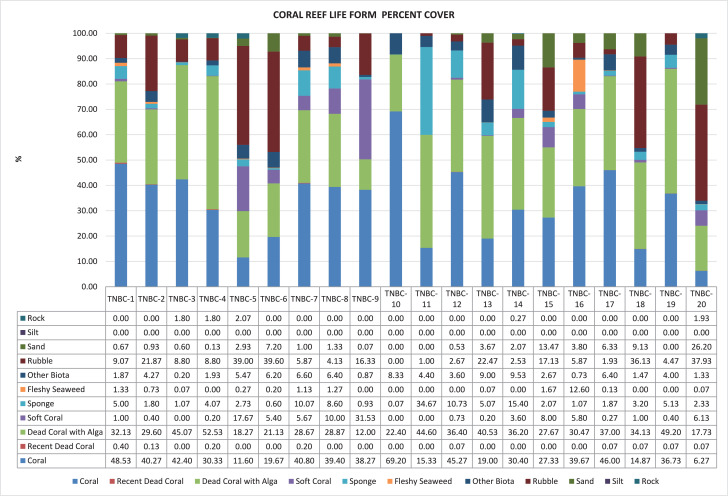
Coral Reef Life Form Percent Cover.

**Fig. 3:**
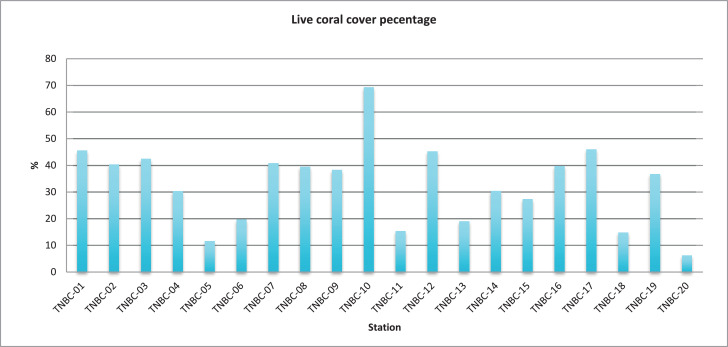
Live coral cover pecentage.

The data reported sampling point (Figure 1), coral reef life form percentage in small islands (Figure 2), Live coral cover percentage (Figure 3), and Station; Geographical Coordinates; Local Name; Live Coral (%) (Table 1). Every island has 4 sampling point with 200 quadrants, totally 20 sampling point with 1000 quadrants. The method used underwater photo transect and Coral Point Count with excel (CPCe) analysis. The average of live coral cover on the five islands is 32.92% with category moderate [Table 1]. Percentage of coral cover between 6,27% and 69,20% (Figure 3). Station TNBC-10 has the highest value for the percentage of coral cover (69,20%) and Station TNBC-20 has the lowest value for the percentage of coral cover (6,27%) (Figure 2). Manado Tua Island has highest percentage of coral cover 42,02%, Bunaken Island 39,63%, Mantehage Island 29,10%, Siladen island 27,87%, and the lowest was Nain Island 25,97% (Table 1). Station TNBC-4 has the highest value for the percentage of dead coral with algae (52,53%) and Station TNBC-9 has the lowest value for the percentage of dead coral with algae (12.00%) (Figure 2).

**Table 1:** Station; Geographical Coordinates; Local Name; Live Coral (%).

Station	Geographical Coordinates		Local Name	Live Coral (%)	Average (%)	Island
	Latitude	Longitude				
TNBC-01	01°36′43,95"	124°47′00,73"	Bunaken Timur 1	45.53	39.63	Bunaken
TNBC-02	01°36′42,92"	124°44′21,56"	Bunaken Muka Kampung	40.27		
TNBC-03	01°36′58,47"	124°45′03,79"	Bunaken Fukui	42.40		
TNBC-04	01°36′11,50"	124°46′58,91"	Bunaken Alungbanua	30.33		
TNBC-05	01°37′37,71"	124°48′04,27"	Siladen Belakang	11.60	27.87	Siladen
TNBC-06	01°37′39,44"	124°47′58,71"	Siladen Timur	19.67		
TNBC-07	01°37′45,76"	124°48′27,70"	Siladen Resort	40.80		
TNBC-08	01°38′12,10"	124°48′21,87"	Siladen Jetty	39.40		
TNBC-09	01°38′07,10"	124°43′05,96"	Manado Tua Pangalingan 1	38.27	42.02	Manado tua
TNBC-10	01°37′57,18"	124°43′04,44"	Manado Tua Pangalingan 2	69.20		
TNBC-11	01°36′58,78"	124°41′34,10"	Manado Tua Bualo 1	15.33		
TNBC-12	01°36′57,50"	124°41′49,04"	Manado Tua Bualo 2	45.27		
TNBC-13	01°41′44,23"	124°45′10,25"	Mantehage Bango	19.00	29.10	Mantehage
TNBC-14	01°41′40,47"	124°46′47,72"	Mantehage Tinongko	30.40		
TNBC-15	01°43′46,08"	124°46′26,03"	Mantehage Buhias	27.33		
TNBC-16	01°42′32,96"	124°47′01,23"	Mantehage Bella Point	39.67		
TNBC-17	01°47′03,39"	124°45′57,62"	Nain Batu Kapal	46.00	25.97	Nain
TNBC-18	01°45′42,78"	124°46′37,89"	Nain Jalan Masuk	14.87		
TNBC-19	01°46′12,42"	124°49′09,13"	Nain Selatan	36.73		
TNBC-20	01°45′33,61"	124°47′34,56"	Nain Pasir Timbul	6.27		
Average				32.92		

## 2 Experimental Design, Materials, and Methods

Twenty visits were orginezed in Februari – March 2020 by boat to five small Islands (Bunaken Island; Siladen Island; Manado Tua Island; Mantehage Island; Nain Island) Bunaken National Park, North Sulawesi Province, Indonesia. The UPT method utilizes advanced technology, both the development of digital camera technology and computer software technology. The underwater photos collected are analyzed using computer software to obtain quantitative data. Advantage in using the UPT method among other is to shorten diving time [1]. Besides, the photos can be used as documentation archieve which can be reviewed anytime. However, there are some weaknesses out of the method, including dependency on the camera and longer time for photo analysis, especially when using technique of area calculation. Data collecting from the field is done by diving using SCUBA diving apparatus. The process is using UPT (Underwater Photo Transect) method utilizing underwater camera or specifically water protected digital camera (housing) to hold sea water seeping. The photo collection of the reef utilizing UPT method consist of underwater photo data along 50 meter transect line with 1 meter interval [1]. The number of photo data are abundant and require higher capacity of databank to preserve. If such data are not very well managed, the photos may unintentionally be exchanged between different research stations. On this acccount, they should be properly handled by way of transferring the preserved files in the memory card into separate databank (external harddisc). It is important to secure out data since the camera might be broken at any time during underwater operation and may cause damage on the camera memory card [1]. The data photos are analyzed to retrieve quantitative data such as percent cover of each benthic categories or substrates. Such data photos can be analyzed using a number of softwares, which are Sigma Scan Pro, Image J or CPCe. In order to obtain quantitative data based on underwater photos resulted from the UPT method, the analysis can be undertaken against each frame by way of selecting number of random point to be used to analyze the photos. The number of random point used is 30 for each frame, and this is adequatly representative to estimate the percentage of benthic categories and substrates [1]. Total of 1000 sample were colected using quadrants with underwater photo transect method. The sample were then analyzed and identified using lab marine biology in Faculty of Fisheries and Marine Science, Sam Ratulangi University, Manado, Indonesia with CPCe analysis. The analysis and identification was undertaken using recommendation suggested by [1,2].

## Declaration of Competing Interest

The authors declare that they have no known competing financial interests or personal relationships which have, or could be perceived to have, influenced the work reported in this article.
